# Identification of Candidate Coral Pathogens on White Band Disease-Infected Staghorn Coral

**DOI:** 10.1371/journal.pone.0134416

**Published:** 2015-08-04

**Authors:** Sarah A. Gignoux-Wolfsohn, Steven V. Vollmer

**Affiliations:** Marine Science Center, Northeastern University, Nahant, Massachusetts, United States of America; King Abdullah University of Science and Technology, SAUDI ARABIA

## Abstract

Bacterial diseases affecting scleractinian corals pose an enormous threat to the health of coral reefs, yet we still have a limited understanding of the bacteria associated with coral diseases. White band disease is a bacterial disease that affects the two Caribbean acroporid corals, the staghorn coral *Acropora cervicornis* and the elkhorn coral *A*. *palmate*. Species of *Vibrio* and *Rickettsia* have both been identified as putative WBD pathogens. Here we used Illumina 16S rRNA gene sequencing to profile the bacterial communities associated with healthy and diseased *A*. *cervicornis* collected from four field sites during two different years. We also exposed corals in tanks to diseased and healthy (control) homogenates to reduce some of the natural variation of field-collected coral bacterial communities. Using a combination of multivariate analyses, we identified community-level changes between diseased and healthy corals in both the field-collected and tank-exposed datasets. We then identified changes in the abundances of individual operational taxonomic units (OTUs) between diseased and healthy corals. By comparing the diseased and healthy-associated bacteria in field-collected and tank-exposed corals, we were able to identify 16 healthy-associated OTUs and 106 consistently disease-associated OTUs, which are good candidates for putative WBD pathogens. A large percentage of these disease-associated OTUs belonged to the order *Flavobacteriales*. In addition, two of the putative pathogens identified here belong to orders previously suggested as WBD pathogens: *Vibronales* and *Rickettsiales*.

## Introduction

Over the past few decades, coral reefs have experienced an unprecedented rise in the prevalence and impacts of coral disease epizootics [1, 2] with especially severe impacts on Caribbean coral reefs [3]. In spite of these impacts and a significant increase in scientific research, we still lack critical information about the etiology and ecology for most of the ~20 described coral diseases [4–6]. The need for a better understanding of coral diseases has escalated with an increasing amount of data tying the rise in coral disease prevalence and increased pathogen virulence to warming ocean temperatures [2, 7].

A major roadblock for coral disease research is the difficulty of isolating and culturing coral disease pathogens [8, 9]. As a result, the discovery of putative pathogens has relied heavily on identifying bacteria that are strongly associated with diseases using genetic techniques such as clone-based 16S rRNA gene sequencing [10–23] and, more recently, high-throughput 16S rRNA gene sequencing and high-throughput microarrays [24–29]. High-throughput 16S rRNA gene sequencing of the coral microbiome has revealed a higher diversity of bacteria than previously thought; in a survey of 16S gene sequencing studies across seven Caribbean coral species, Sunagawa et al. (2009) predict that each individual coral harbors several thousand operational taxonomic units (OTUs). This high diversity makes identifying putative pathogens in the coral microbiome more difficult.

One of the most destructive coral diseases to date is white band disease (WBD), a host-specific disease that affects the two Caribbean acroporid species: *Acropora cervicornis* (staghorn coral) and *A*. *palmata* (elkhorn coral). Since it was first observed in 1979 [30], WBD has caused unprecedented Caribbean-wide mass die-offs of these critical reef-building species [31], resulting in the recent listing of both species as endangered under the US Endangered Species Act [32]. WBD is infectious and can be transmitted by direct contact between corals, through the water column to an injured coral, and by the corallivorous snail *Coralliophila abbreviata* [33, 34]. Previous work confirms that WBD is caused by a bacterial pathogen [35, 36] and multiple putative pathogens have been identified [17, 36–38]. Ritchie and Smith (1998) isolated a strain of *Vibrio* from WBD-infected *A*. *cervicornis* that was similar to *Vibrio charchariae* in both metabolism and morphology [37, 39] and Gil-Agudelo et al. (2006) were then able to elicit WBD signs in a healthy coral with a putative pathogen similar to *V*. *charchariae* [38]. Sweet et al. (2014) used a combination of culture independent and culture dependent antibiotic experiments to identify three candidate WBD pathogens: *V*. *charchariae*, *Lactobacillus suebicus*, and *Bacillus sp*. Casas et al. (2004) used culture-independent 16S rRNA sequences to identify a novel *Rickettsiales*-like bacterium associated with *A*. *cervicornis* fragments that were collected after the outbreak of WBD, but which was absent from museum samples collected prior to the outbreak. Because independent studies have identified different putative WBD pathogens, it is possible that multiple pathogens (either singularly or in combination) could elicit WBD signs.

In this study, we used Illumina 16S rRNA gene sequencing to compare the bacterial communities of healthy and diseased (WBD infected) *A*. *cervicornis* collected in the field from four sites in two different years. We then conducted tank-based infection experiments to identify differences in the bacterial communities of infected, exposed but asymptomatic, and healthy control corals, and compared these data to the two years of field data. Non-metric multidimensional scaling (nMDS) and PERMANOVA were used to characterize community-level changes in the coral microbiome due to disease state, site, and year. Multi-factor negative binomial generalized linear models (GLMs) were then used to quantify significant changes in individual OTU abundances between diseased and healthy corals in the field and tank datasets. Those OTUs that were strongly and consistently associated with disease in both datasets are the most likely WBD pathogens.

## Materials and Methods

### Field collections

Collection permits were provided by Autoridad Nacional del Ambiente (ANAM#SE/A-1-12) for sampling of the protected species *Acropora cervicornis*. Seventy-nine one cm fragments of *A*. *cervicornis* (30 healthy and 49 diseased) were collected from the field in the summers of 2009 and 2010 from four sites in Coral Cay, Bocas del Toro, Panama approximately 500 m apart. WBD interfaces were tagged with cable ties prior to collection and only samples with actively progressing WBD were sampled from the field. Diseased coral samples were cut at the interface of living tissue and dead skeleton. Healthy samples were taken from completely asymptomatic coral colonies. Corals were transported from the field in separate containers and placed in one ml of DNA buffer for preservation [40]. Seventy-nine samples were extracted: 49 diseased and 30 healthy. Numbers of corals collected from each site are available in S1 Table.

### Tank-based infection experiment

In February of 2012, 36 healthy five cm fragments of *A*. *cervicornis* from six colonies were collected, transported back to the wet lab at the Smithsonian Tropical Research Institute, and cable-tied to plastic louver. One fragment from each colony (six total fragments/tank) was randomly placed in six flow-through 20 L aquaria with a koralia nano powerhead (Hydor USA Inc., Sacramento, CA, USA) to acclimate for one day. A disease homogenate was made by first vortexing six five cm coral fragments with active WBD signs in separate falcon tubes with glass beads and 15 mL filtered seawater (after Kline and Vollmer 2011) and then pooling together the separate homogenates. A healthy (control) homogenate was made in the same manner using coral fragments from colonies that did not show signs of disease. Flow was stopped and three tanks were each dosed with 30 mL of diseased coral homogenate and the other three were dosed with 30 mL of the healthy homogenate. Healthy coral fragments were then monitored every two hours for disease signs. As infected corals exhibited signs of WBD (after 40 hours), they were removed from the experimental tanks and one cm of coral tissue at the disease interface was preserved in DNA buffer. Healthy fragments were all sampled on day four of the experiment. Number of corals infected in each tank is available in S2 Table. Total DNA was extracted from 19 fragments from this experiment: seven dosed with disease homogenate and contracted WBD (DD), seven were dosed with healthy homogenate and remained healthy (HH), and five were dosed with disease homogenate but remained healthy or asymptomatic (DH).

### 16S library preparation

DNA was extracted from samples preserved in guanidine thiocyanate DNA buffer [40] using the Agencourt DNAdvance bead extraction kit (Agencourt Bioscience Corporation, Beverly, MA, USA) a blank DNA extraction was performed with each round of extractions. The V6 hypervariable region of the ribosomal small subunit 16S gene was chosen as the target due to its short length but high sensitivity to diversity, making it a good candidate for Illumina sequencing which has short read lengths but high sequencing depth [41–43]. The V6 region was amplified with custom barcoded primers that consist of a region that anneals to the V6 region of interest, followed by a unique five basepair barcode, and the Illumina sequencing adapter [44]:

V6-L [5’-CGGTCTCGGCATTCCTGCTGAACCGCTCTTCCGATCTnnnnnACRACACGAGCTGACGAC-3’]

V6-R [5’-CGGTCTCGGCATTCCTGCTGAACCGCTCTTCCGATCTnnnnnACRACACGAGCTGACGAC-3’]

Barcodes differed by two or more basepairs to reduce incorrect barcode calling. A separate, 40 μl PCR reaction for each sample was performed with a unique combination of primers: 5 μl each 4mM primer, 8μl standard Taq buffer (New England Biolabs, Ipswich, MA, USA), 0.8μl dNTPs, 20 μl diH20, 0.5 μl Taq DNA polymerase (NEB) for the following cycle: 94°C for 2m, 28 cycles of: 94°C for 15s, 55°C for 15s, 72°C for 30s, followed by 72°C for 1m. A negative control and blank was amplified with each set of reactions. PCR reactions were pooled and amplified with the Illumina primers: OLJ139[5’AATGATACGGCGACCACCGAGATCTACACTCTTTCCCTACACGA3’] OLJ140 [5’CAAGCAGAAGACGGCATACGAGATCGGTCTCGGCATTCCTGCTGAAC3’] in a 40 μl reaction: 8 μl Phusion buffer (NEB) 0.8 μl dNTPs, 0.5 μl Phusion HIfidelity Taq (NEB), 20.2 μl diH20, 0.5 μl DNA (previous PCR product), for the following cycle: 98°C for 2m, 12 cycles of: 98°C for 1m, 55°C for 1m, 72°C for 1m, and finally 72°C for 5m. Final PCR products were cleaned using the DNAmpure beads (Agencourt), concentration and length were verified using the Agilent 2100 Bioanalyzer system (Agilent, Santa Clara, CA, USA) and sequenced using paired-end 150 basepair sequencing on the Illumina Hiseq 2000 at Tufts University.

### Bioinformatics

Paired reads were overlapped using FLASH [45]. Basepairs with a Phred score <q20 were trimmed. Sequences were assigned sample IDs using a custom Python script (available at: https://github.com/eholum/BioStuffs). Samples were then run through QIIME v.1.7.0 [46]. Briefly, OTUs were chosen based on 97% sequence identity using the open reference OTU picking workflow using UCLUST against the Greengenes database [47]. Taxonomy of each OTU was assigned using BLAST against the Greengenes database and those samples that corresponded to chloroplast DNA in order or lower taxonomic levels were excluded as algal symbiont contamination. OTU counts were then normalized using the size factors method from the R package DESeq2 [48] prior to statistical analyses to account for differences in library size (an alternative to rarefaction) and OTUs observed fewer than 10 times were removed in order to reduce undue bias of low abundance OTUs. Statistical analyses were run both with and without low abundance OTUs and results did not change significantly.

### Statistical analyses

Dissimilarity between samples was visualized using non-metric multidimensional scaling (nMDS) using the metaMDS function in the R package Vegan [49], and community level differences between the three factors (disease state, site, year) were tested by PERMANOVA of Bray-Curtis dissimilarities using the Adonis function and by fitting the factors to the nMDS using the function envfit. Diversity metrics were calculated using the functions rarefy and diversity in Vegan. Significant differences in the abundance of individual OTUs were identified using a multifactorial negative binomial GLM, implemented in the R package DESeq2 [48]. The data were fit to a negative binomial distribution and significantly different OTUs (p-value adjusted by FDR <0.05) between diseased and healthy samples were determined using the Wald test for significance of GLM terms [48]. Analyses were run separately for the field and tank-exposed data. In the field data, two separate GLMs were run for site (disease state x site) and year (disease state x year) and OTUs that differed significantly in either were considered to be transient OTUs. Disease-associated OTUs were compared between both datasets to identify OTUs with consistent and strong associations to WBD. Transient OTUs that were present in the list of disease-associated OTUs were placed in a second tier of disease-associated OTUs, leaving only those OTUs that were consistently disease-associated and did not differ due to site or year in tier 1. R scripts for statistical analyses are available at https://github.com/sagw/R-scripts.

### Nucleotide sequence accession numbers

Sequences from this study have been deposited in the SRA database under accession numbers SRR2078055-SRR2078108

## Results

16S v6 rRNA gene sequencing from the field-collected corals yielded 10,849,364 reads across 79 samples (49 diseased and 30 healthy). The data from tank-exposed corals contained 2,954,558 reads across 19 samples (seven exposed to disease that contracted disease (DD), seven exposed to healthy that remained healthy (HH), and five exposed to disease that remained healthy (DH)). Read length varied from 57–137 bp. After clustering reads from both datasets at 97% similarity, 43,304 OTUs were identified. The field data contained 32,347 OTUs while the tank infection data had 18,195 OTUs. In all, 7,408 of these OTUs were shared between the two datasets. In both datasets, diseased corals exhibited elevated OTU diversity and richness, but OTU diversity was only significantly higher in diseased corals from the field (Table 1).

**Table 1:** Diversity of OTUs. Bolded text indicates significantly different values as determined by Welch’s two sample t-test.

	Field		Tank		
	D	H	DD	DH	HH
Number of samples	49	30	7	5	7
Number of OTUs	26,579	9,240	11,754	5,821	5,493
Simpson Diversity	**0.84**	**0.64**	0.89	0.87	0.78
Shannon diversity	**3.08**	**2.36**	3.77	3.29	2.82
Rarefied richness	542.42	308.00	1,679.14	1,164.20	784.71

PERMANOVA detected strong, community-level differences in the coral bacterial communities in the field as well as in the tank-exposed corals. In the field-collected corals, the coral microbiome differed significantly due to disease state (R^2^ = 0.10, p = 0.001), site (R^2^ = 0.12, p = 0.001, and year (R^2^ = 0.019, p = 0.011) as well as all combinations of interactions (Table 2). Disease state and site had similar and strong levels of influence on the bacterial community (R^2^ of 0.10 and 0.12, respectively). To confirm the effects of the primary factors (disease state, site, and year) on OTU abundance, the factors were fit to the nMDS using envfit. Disease state (R^2^ = 0.39, p = 0.001) and site (R^2^ = 0.26, p = 0.001) had significant effects, with disease state having a greater influence on the bacterial community. These results corroborate the strength of the effects of site and disease state on the bacterial communities as demonstrated by PERMANOVA, the differences are most likely due to the reduction in dimensional space by nMDS.

**Table 2:** Results of PERMANOVA based on Bray-Curtis dissimilarities of the relative abundance of OTUs on field-collected *A*. *cervicornis* in response to disease state, site, and year.

	Df	SumsOfSqs	MeanSqs	F.Model	R2	Pr(>F)
Disease_state	1	3.22	3.22	10.96	0.10	0.001
Site	3	3.78	1.26	4.27	0.12	0.001
Year	1	0.617	0.62	2.09	0.019	0.011
Disease_state:Site	3	1.60	0.53	1.81	0.05	0.001
Disease_state:Year	1	0.68	0.68	2.32	0.022	0.005
Site:Year	3	1.74	0.58	1.98	0.06	0.001
Residuals	64	18.83	0.29	NA	0.60	NA
Total	78	31.13	NA	NA	1	NA

**Fig 1:**
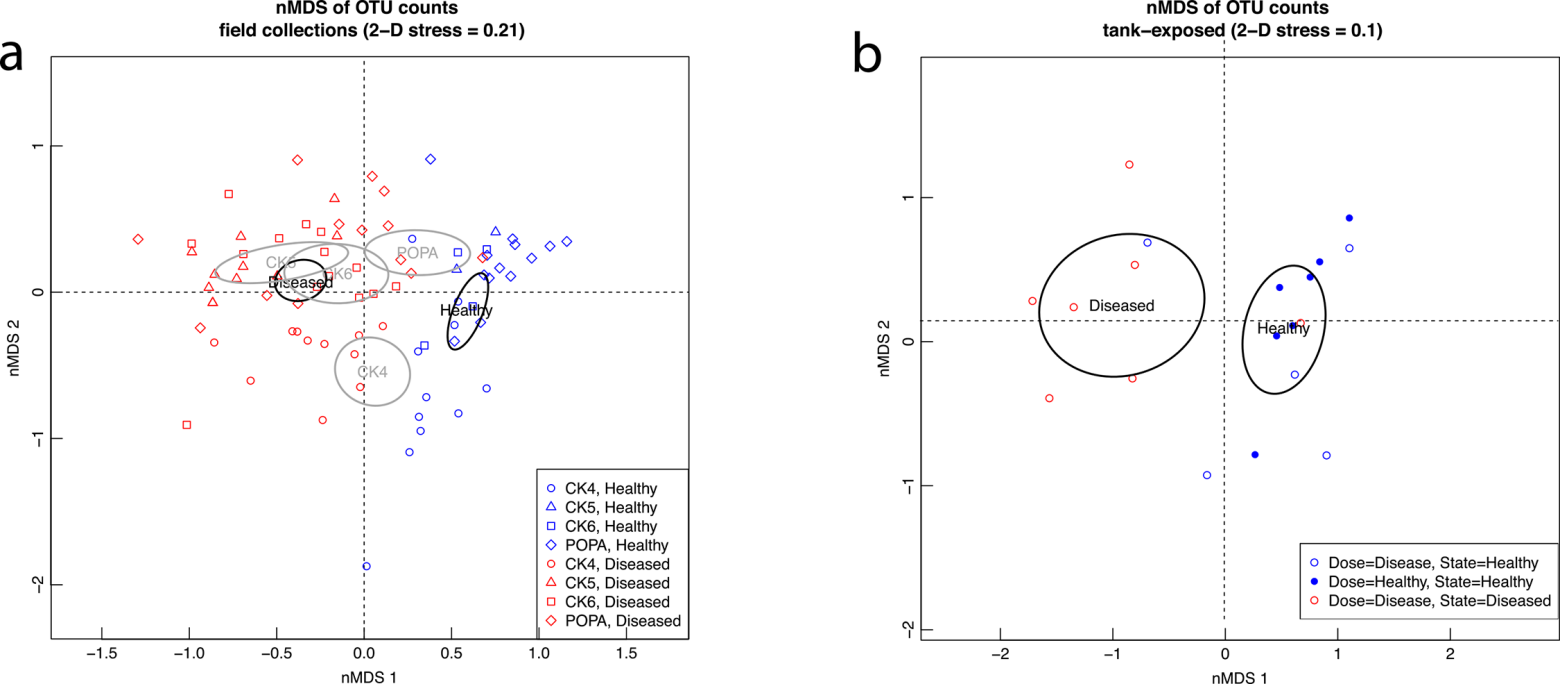
nMDS plots of dissimilarities between samples (a) Field collection samples, showing clustering according to disease state. “Healthy” “Diseased” and site names denote the centroids of each group and ellipses are 95% confidence ellipses. (**b)** Tank-exposed samples, showing clustering according to disease state. “Healthy” and “Diseased” labels denote the centroids of each disease state and ellipses are 95% confidence ellipses.

On the nMDS plot, diseased and healthy corals separated along nMDS1 while corals separated by site along nMDS2 (Fig 1A). PERMANOVA and nMDS analyses using presence/absence data (not shown) gave the same results as the abundance data, indicating that significance was not driven solely by differences in abundance of OTUs.

To further explore the strength of the effects of site on diseased and healthy corals, we split the data from diseased and healthy corals and ran two separate PERMANOVAs. These PERMANOVAs indicated that site had a larger effect on the bacterial communities of healthy corals (R^2^ = 0.28, p = 0.001, Table 3) than on those of diseased corals (R^2^ = 0.13, p = 0.001, Table 4).

**Table 3:** Results of PERMANOVA based on Bray-Curtis dissimilarities of the relative abundance of OTUs on field-collected healthy *A*. *cervicornis* in response to site and year.

	Df	SumsOfSqs	MeanSqs	F.Model	R2	Pr(>F)
Site	3	2.80	0.93	3.69	0.28	0.001
Year	1	0.54	0.54	2.13	0.054	0.041
Site:Year	2	0.73	0.37	1.45	0.074	0.10
Residuals	23	5.82	0.25	NA	0.58	NA
Total	29	9.89	NA	NA	1	NA

**Table 4:** Results of PERMANOVA based on Bray-Curtis dissimilarities of the relative abundance of OTUs on field-collected diseased *A*. *cervicornis* in response to site and year.

	Df	SumsOfSqs	MeanSqs	F.Model	R2	Pr(>F)
Site	3	2.35	0.78	2.40	0.13	0.001
Year	1	0.79	0.78	2.41	0.042	0.001
Site:Year	3	1.36	0.46	1.39	0.074	0.018
Residuals	43	14.04	0.33	NA	0.76	NA
Total	50	18.54	NA	NA	1	NA

PERMANOVA on the dataset from tank-exposed corals also indicated a significant effect of disease state (R^2^ = 0.29, p = 0.007), which was supported by the nMDS plot, where diseased and healthy corals separated along nMDS1 (Fig 1B). Interestingly, the corals that were exposed to disease but remained asymptomatic (DH) clustered more closely on the nMDS plot with healthy (HH) corals than diseased (DD) corals.

**Fig 2:**
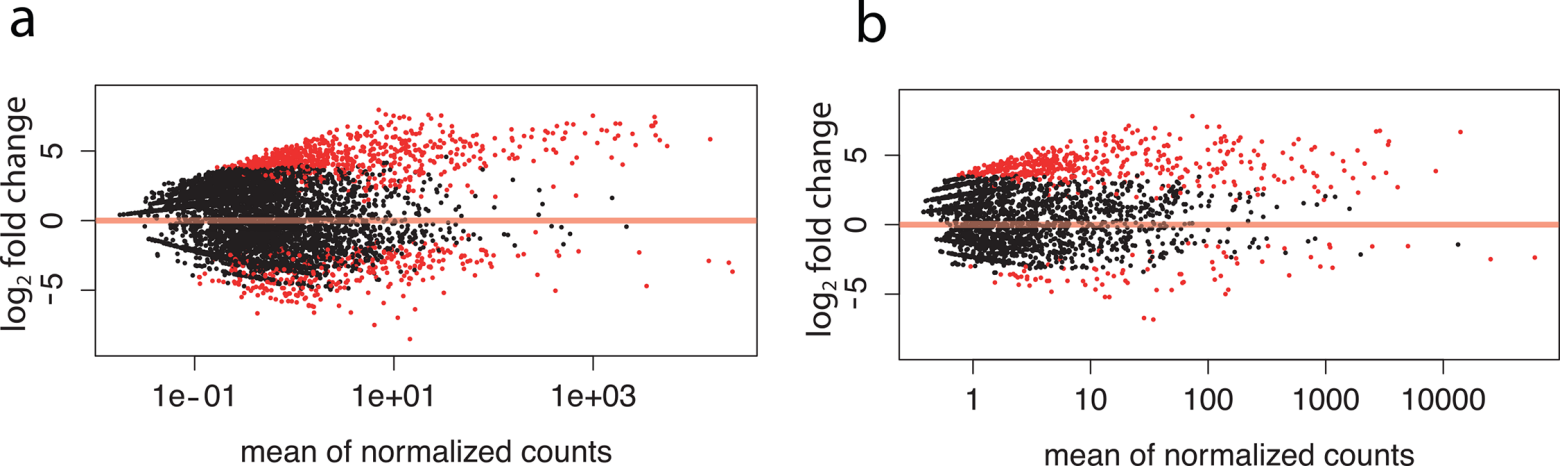
Plots of the log2 fold abundance change of each OTU by the mean of normalized counts. Significantly more or less abundant OTUs are in red (**a**) Field-collected corals by year + disease state (**b**) Tank-exposed corals by final disease state.

The negative binomial GLM on the field data comparing the abundance of OTUs across disease state and year identified 1,363 individual OTUs that differed significantly due to disease state, 20 OTUs that differed significantly due to year, and 66 OTUs that differed significantly due to the interaction of disease state and year (Table 5). The majority of the OTUs that differed due to disease state (1,012 or 74%) were significantly more abundant on diseased corals than healthy corals (Fig 2A). In the tank-infected corals, the negative binomial GLM comparing disease-exposed corals that contracted disease (DD) with healthy-exposed (i.e. control) corals (HH) identified 521 OTUs associated with disease state, the majority of which (n = 494; 95%) were more abundant on the disease-infected corals (Fig 2B).

**Table 5:** Significantly associated OTUs in tank and field-collected datasets.

Dataset	Factor	Level	Significantly associated OTUs
Field	Disease state		1,363
		Diseased	1,012 (74%)
		Healthy	351 (26%)
	Year		20
	Disease state * Year		66
Tank	Disease State		521
		Diseased	494 (95%)
		Healthy	27 (5%)

**Fig 3:**
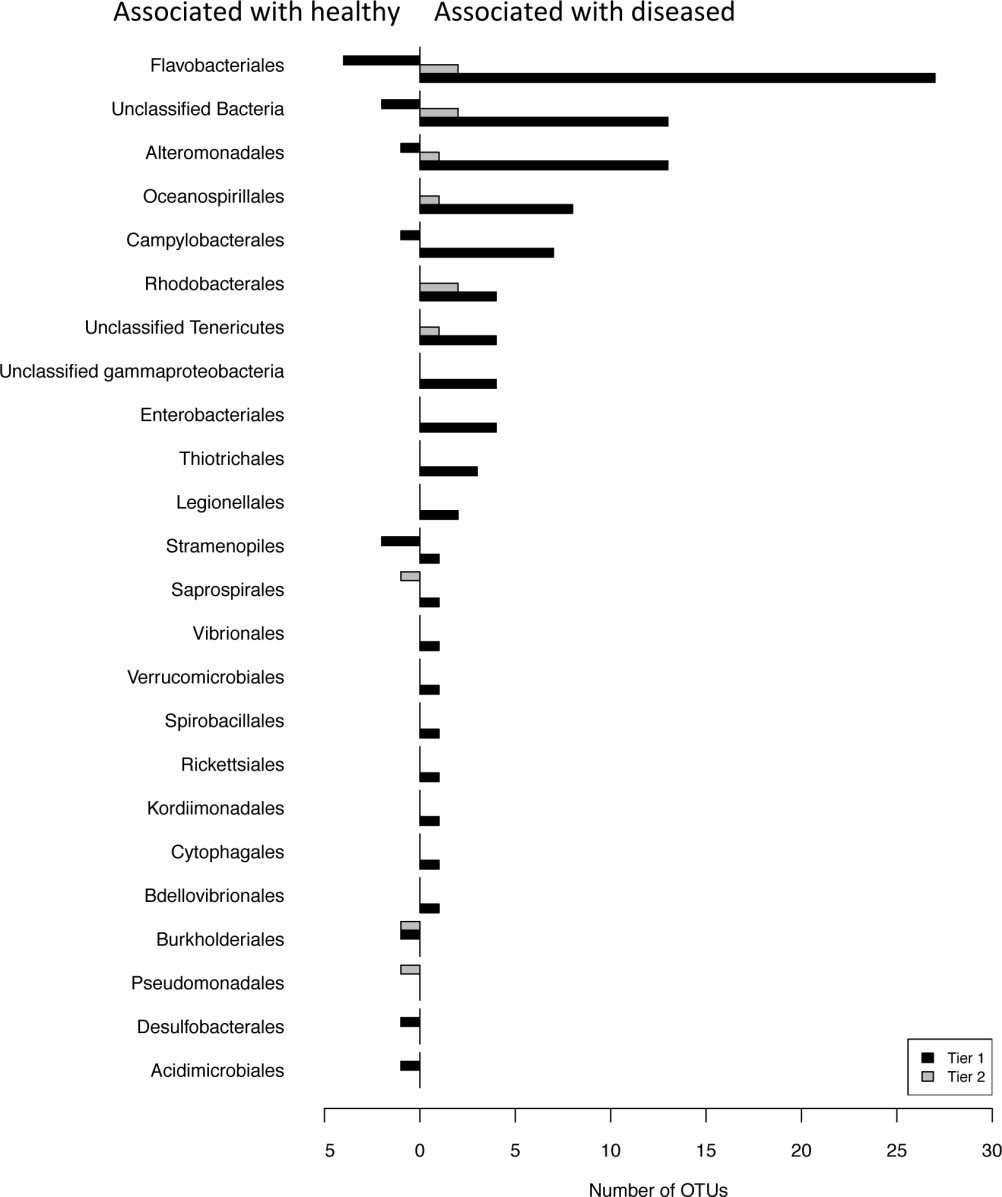
Taxonomic classification on the level of order of OTUs that are significantly more or less abundant in diseased corals compared to healthy across both tank-exposed and field-collected corals. Tier 1 consists of those OTUs that did not differ significantly across site and year, tier 2 consists of OTUs that differed significantly due to disease state and site and/or year.

By comparing disease and healthy-associated OTUs in the field-collected dataset to those in the tank-exposed dataset, we identified 106 consistently disease-associated OTUs (i.e. more abundant in diseased corals) and 16 consistently healthy-associated OTUs (i.e. more abundant in healthy corals) and classified them by order (Fig 3). We further narrowed the list of consistently disease- or healthy-associated OTUs by dividing them into two tiers: tier 1 consists of those OTUs that differed significantly by disease state but not by site or year, and tier 2 consists of those OTUs that differed by disease state as well as by site or year (i.e. were significantly different in the DESeq model ~disease state:site or ~disease state:year). We identified 12 tier 2 OTUs: seven associated with diseased corals and two associated with healthy corals. Disease-associated tier 2 OTUs consisted of seven OTUs that differed by year (two belonging to *Flavobacteriales*, two *Rhodobacterales*, one *Alteromonadales*, one *Oceanospirillales*, one unidentified) and two OTUs that differed by both year and site (one belonging to the phylum *Tenericutes* and one unidentified). Healthy-associated tier 2 OTUs consisted of two OTUs that differed by site (belonging to the orders *Burkholderales* and *Pseudomonadales*) and one OTU that differed by both year and site (*Saprospirales*).

The five bacterial orders with the greatest number of disease-associated OTUs in tier 1 were: *Flavobacteriales* with 27 OTUs, *Alteromonadales* with 13 OTUs, *Oceanospirillales* with eight OTUs, *Campylobacterales* with seven OTUs, and *Rhodobacterales* with four OTUs (Fig 3). The three bacterial orders with multiple healthy-associated OTUs were: *Flavobacteriales* with four OTUs and *Stramenopiles* with two OTUs. Complete taxonomic information for all significantly different OTUs can be found in S3 Table.

## Discussion

When *Acropora cervicornis* is infected with white band disease, its microbiome changes drastically, with an increase in hundreds of disease-associated OTUs, rather than just a few potential pathogens. The shift from a healthy to a diseased coral microbiome was marked by elevated microbial diversity and richness, a pattern that is consistent across other coral diseases [14, 19, 23–25, 28, 50]. We also detected strong site-specific variation in the microbiomes of all corals. Interestingly, the site-specificity of the bacterial communities was strongest on healthy corals, and thus contraction of WBD appears to reduce this site specificity. Because we identified a large number of disease-associated OTUs, the observed differences in the bacterial communities of diseased and healthy corals are not likely the results of a change in the abundance of a single primary pathogen, but are also due to changes in opportunistic pathogens, secondary colonizers, and saprophytic bacteria. Many of the OTUs that are more abundant on diseased corals are also present on healthy corals. This could be because some apparently ‘healthy’ corals are asymptomatic, but harbor subclinical infections of disease-causing bacteria. Alternatively, these OTUs may be ‘pathobionts’[51]: microbes that are present on the host but become pathogenic in certain hosts due to environmental conditions or host susceptibility. In all, we identified 97 consistently disease-associated bacteria across space and time belonging to 17 different orders of bacteria; the most abundant and noteworthy are discussed below.

*Flavobacteriales* made up the largest percentage (27 OTUs, 27%) of our disease-associated OTUs and are therefore likely involved in the WBD etiology. *Flavobacteria* are well-known pathogens of fish [52, 53]. White band disease shares some notable characteristics with flavobacterial diseases in fish. Two common flavobacterial fish pathogens, *Flavobacteria psychrophilum* and *F*. *columnaris*, both cause open lesions and tissue necrosis [52–54], similar to WBD. Furthermore, infection of salmonids with *F*. *columnaris* can be characterized by simultaneous infection with other strains of *Flavobacteria* [55], suggesting that one of the 27 WBD-associated *Flavobacteriales* OTUs identified here may be the primary pathogen. The similarities between WBD and columnaris disease are strengthened by our finding that *Pseudomonadales*, which is an antagonist of *F*. *columnaris* [55], was consistently associated with healthy corals. Lower abundances of *Pseudomonadales* in an initially healthy coral may allow strains of *Flavobacteria* to infect and proliferate and thereby cause disease. If the etiology of WBD is in fact similar to that of columnaris disease, then one of the 27 *Flavobacteriales* OTUs that are associated with WBD may be a keystone pathogen. Keystone pathogens cause shifts in the natural bacterial community of their host, which lead to a diseased state, making it harder to detect the primary pathogen [56, 57]. Because *Flavobacteriales* are also found on healthy corals, they may be pathobionts or opportunistic pathogens. In addition, multiple strains of *Flavobacteriales* may elicit the same characteristic disease signs in *A*. *cervicornis*, or they may work together to cause WBD. Understanding the time-course of infection with strains of *Flavobacteriales* will help to elucidate what roles the different flavobacterial species are playing in the diseased coral. *Flavobacteria* have been largely overlooked as potential pathogens in coral diseases, yet species of *Flavobacteria* have been previously associated with both black band disease and white plague disease [10, 22, 24, 58]. *Flavobacteria* may therefore be more important in coral disease as a whole than previously thought.

The next most abundant orders of disease-associated OTUs—*Alteromonadales* (13 OTUs), *Oceanospirillales* (eight OTUs), *Campylobacterales* (eight OTUs), and *Rhodobacterales* (four OTUs)—are not likely candidates for a primary WBD pathogen. While they have all been associated with corals and in some cases coral diseases, none of these orders include well-characterized coral pathogens, but instead seem to play either a beneficial, opportunistic, or secondary role in the diseased coral. *Alteromonadales* have been associated with both white plague disease [24, 25], and yellow band disease [19], but have also been identified as “resident” coral bacteria [59–62]. The only known pathogen in this order is *Thalassomonas loyana*, the causative agent of a white plague-like disease, but members of the *Alteromonadales* order are not well-known pathogens in other animals [63]. Members of the order *Oceanospirillales* have not been previously associated with any coral or other animal diseases [4, 64–66], but have been recently been shown to be commonly associated with corals potentially as beneficial symbionts [50, 67]. Some members of *Oceanospirillales* are well-known for their role in the bacterial bloom following the Deepwater Horizon oil spill [68, 69] and their ability to degrade Dimethylsulfoniopropionate, hydrocarbons, and amino acids [70–72]. Members the order *Campylobacterales* are zoonotic pathogens which are commensal in marine mammals and birds [73, 74]. While strains of *Campylobacterales* have been associated with coral diseases previously, including black band disease [10], white syndrome, brown band disease [21], and white plague [24], their role in the etiologies of these diseases remains unclear. Given that *Campylobacter* are frequently found in sewage [75], they may be introduced to the coral microbiome via human sewage deposition into the ocean. *Rhodobacteraceae*, our fifth most common disease-associated order, have also been associated with many coral diseases in multiple species across a variety of locations [4, 24, 25, 76], but have not been identified as a pathogen in corals or other animals. One of the six disease-associated *Rhodobacteraceae* identified here was present in every diseased and healthy coral sampled across both datasets, but increased in abundance on diseased corals, similar to previously described changes in abundance of strains of *Rhodobacter* in response to disease in other corals [24]. The disease-associated OTUs belonging to these four orders are likely either 1) secondary opportunistic colonizers, which take advantage of a diseased coral’s weakened state [24], 2) beneficial symbionts, which increase in an effort to combat the primary pathogen, or 3) saprophytic bacteria, which are attracted to the increased nutrients of a dying coral.

In addition to these bacterial orders with multiple disease-associated OTUs, we identified two previously proposed WBD pathogens among the disease-associated OTUs: one belonging to the family *Vibrionaceae* and one to *Rickettsiaceae*. Species of *Vibrio* have been confirmed as pathogens in other coral diseases (Cervino et al. 2008, Ushijima et al. 2012), most notably *V*. *corallilyticus* in *Pocillopora damicornis* tissue lysis [77]. *Vibrios* are of special interest as pathogens given their well-characterized role in human diseases such as cholera [78]. Ritchie and Smith (1998) first isolated a strain of bacteria from diseased corals that was identified as *Vibrio charchariae* through morphological and metabolic methods. Gil-Agudelo et al. (2006) were then able to isolate and genetically identify a strain of *Vibrio charchariae* from diseased corals in Puerto Rico, which elicited signs similar to WBD in healthy corals [36, 38]. While more research is needed to confirm the relationship of the strain identified here to *Vibrio charchariae*, our identification of a consistently white band disease-associated *Vibrio* using culture-independent methods supports the findings of these previous studies. We also identified five unclassified gammaproteobacteria associated with diseased corals, which may be *Vibrio* and warrant further investigation.

Members of the order *Rickettsiales* are well-characterized pathogens in marine invertebrates [79]. As obligate intracellular parasites, pathogenicity of *Rickettsia* can only be determined using genetic or histological methods, not in culture [80]. *Rickettsia*-like organisms have been associated with diseased Caribbean acroporids in both histological and genetic studies [17, 81]. Casas et al. (2004) identified a coral-associated *Rickettsiales* sequence (called CAR-1a) on *A*. *cervicornis* collected after the outbreak of WBD, which was absent on museum specimens predating disease. CAR-1a was common on both diseased and healthy *A*. *cervicornis*, suggesting that it is likely an opportunistic pathogen, which only becomes pathogenic under certain conditions. Similarly in our study, *Rickettsiaceae* was found on more than 90% of all *A*. *cervicornis* both from the field (92% on healthy; 97% on diseased) and tanks (100% of corals). While more information is needed to determine if the *Rickettsiaceae* observed here is the previously detected CAR-1a strain, it does display similar patterns of abundance.

Given the large number of WBD-associated OTUs, including two previously suggested putative pathogens as well as many strains of *Flavobacteria*, we should consider the possibility that multiple bacterial species could elicit WBD signs either singularly or as a consortium. Coral diseases such as yellow band disease and black band disease are caused by a consortium of bacteria [22, 82]. In WBD, multiple disease-associated bacteria may be able to cause disease signs in slightly different combinations. Alternatively, one or more of these putative pathogens may actually be secondary colonizers, bacteria that are only able to colonize the host after the primary colonizer (or pathogen) has infected and altered the host environment.

When looking at other factors that contribute to coral microbiome variability, we found that site had as large an effect on the composition of the coral microbiome as disease. While the collection sites were only two to six km apart, this finding is similar to the effects of site on the bacterial communities of other species of coral [25, 27, 28, 50, 83, 84]. Interestingly, we found that site had an effect on both healthy and diseased coral bacterial communities. However, site had less of an effect on diseased coral-associated bacterial communities, indicating that WBD reduces the natural site-specificity of coral-associated bacteria. We identified 5 OTUs in our list of disease- or healthy-associated OTUs that differed due to site and disease state belonging to the orders: *Burkholderales*, *Pseudomonadales*, *Saprospirales*, *Tenericutes*, and unidentified bacteria. Further investigation into OTUs that differ due to disease and site will require larger sample sizes from each site. The site-specific variation of diseased coral-associated bacteria is likely complicating our search for primary coral disease pathogens.

In examining WBD-associated bacterial communities, we have identified multiple previously proposed WBD pathogens, and propose strains of *Flavobacteriales* as new putative WBD pathogens. Further work should examine the possibility that multiple strains are involved in causing WBD. To separate the cause of the disease from the effects, the timing of colonization of diseased corals by the disease-associated bacteria must be examined. While culturing of pathogens is still required to confirm etiology, this culture-independent genetic analysis of diseased individuals will greatly inform future culture-based experiments. Understanding diseased and healthy bacterial communities on lower-level organisms such as corals can aid our understanding of more complex bacterial communities such as those in the human gut. Ultimately, a better characterization of coral-associated bacterial communities will inform our quest to understand and mitigate the current rise in coral diseases.

## Supporting Information

S1 Table Number of corals collected from each site. (DOCX)

S2 Table Infection rate for corals in tank-based infection experiment. (DOCX)

S3 Table Significantly different OTUs between diseased and healthy for the field and tank datasets. (XLSX)
