# Correction: Identification of Candidate Coral Pathogens on White Band Disease-Infected Staghorn Coral

**DOI:** 10.1371/journal.pone.0142760

**Published:** 2015-11-06

**Authors:** Sarah A. Gignoux-Wolfsohn, Steven V. Vollmer

There is an error in the third sentence of the “16S library preparation” subsection of the Materials and Methods. The first primer listed is incorrect. The correct sentence is: The V6 region was amplified with custom barcoded primers that consist of a region that anneals to the V6 region of interest, followed by a unique five basepair barcode, and the Illumina sequencing adapter [44]:

V6-L [5’- ACACTCTTTCCCTACACGACGCTCTTCCGATCTnnnnnCWACGCGARGAACCTTACC-3’]

V6-R [5’-CGGTCTCGGCATTCCTGCTGAACCGCTCTTCCGATCTnnnnnACRACACGAGCTGACGAC-3’]
