# Supplementary material for: Identification of Candidate Coral Pathogens on White Band Disease-Infected Staghorn Coral
Source: PLoS One. 2015 Aug 4;10(8):e0134416. doi: 10.1371/journal.pone.0134416 (PMC4524643; doi:10.1371/journal.pone.0134416)
Supplement: S1 Table — (DOCX) [file pone.0134416.s001.docx]

| Disease state | Year | Site | # corals collected |
| --- | --- | --- | --- |
| Healthy |  |  |  |
|  | 2009 |  |  |
|  |  | CK4 | 6 |
|  |  | CK5 | 2 |
|  |  | CK6 | 3 |
|  |  | Popa | 9 |
|  | 2010 |  |  |
|  |  | CK4 | 5 |
|  |  | CK5 |  |
|  |  | CK6 | 1 |
|  |  | Popa | 4 |
| Diseased |  |  |  |
|  | 2009 |  |  |
|  |  | CK4 | 9 |
|  |  | CK5 | 5 |
|  |  | CK6 | 9 |
|  |  | Popa | 9 |
|  | 2010 |  |  |
|  |  | CK4 | 2 |
|  |  | CK5 | 6 |
|  |  | CK6 | 5 |
|  |  | Popa | 4 |

Supplementary Table 1. Number of corals collected from each site
