# Supplementary material for: Identification of Candidate Coral Pathogens on White Band Disease-Infected Staghorn Coral
Source: PLoS One. 2015 Aug 4;10(8):e0134416. doi: 10.1371/journal.pone.0134416 (PMC4524643; doi:10.1371/journal.pone.0134416)
Supplement: S2 Table — (DOCX) [file pone.0134416.s002.docx]

| Dose | Tank | N | # WBD | % WBD | #taken for 16S |
| --- | --- | --- | --- | --- | --- |
| Diseased |  |  |  |  |  |
|  | Tank 1 | 6 | 4 | 67% | 3 |
|  | Tank 2 | 6 | 5 | 83% | 5 |
|  | Tank 3 | 6 | 2 | 33% | 4 |
| Healthy |  |  |  |  |  |
|  | Tank 4 | 6 | 0 | 0% | 4 |
|  | Tank 5 | 6 | 0 | 0% | 2 |
|  | Tank 6 | 6 | 1 | 17% | 1 |

Supplementary Table 1. Infection rate for corals in tank-based infection experiment
